# Adult Aging Moderates the Relationship Between Trait Cognitive Anxiety and Subjective Everyday Cognitive Difficulties

**DOI:** 10.3389/fpsyg.2021.747839

**Published:** 2021-10-28

**Authors:** David M. Spalding, Kerry MacAngus, Martine K. Moen, Louise A. Brown Nicholls

**Affiliations:** School of Psychological Sciences and Health, University of Strathclyde, Glasgow, United Kingdom

**Keywords:** aging/ageing, older adults, anxiety, perception, cognition, memory, attention, language

## Abstract

The present aim was to determine, across the adult lifespan, the extent to which different dimensions of trait anxiety might affect subjective cognitive difficulties in everyday life. Following Attentional Control Theory (ACT; Eysenck et al., 2007), we predicted that trait anxiety would have a greater effect on attention and verbal abilities than on visual abilities. We also expected trait cognitive anxiety to exhibit more robust relationships with cognition than trait somatic anxiety. Importantly, we predicted that effects of anxiety would be greater in older adults, in line with the Strength and Vulnerability Integration model (SAVI; Charles, 2010). The sample comprised 286 United Kingdom-based adults aged 18–93 years. Participants completed self-report measures of trait cognitive and somatic anxiety (the State-Trait Inventory for Cognitive and Somatic Anxiety; STICSA, Ree et al., 2008) and everyday cognitive difficulties (the Multiple Abilities Self-Report Questionnaire; MASQ, Seidenberg et al., 1994). Moderated regression models were constructed, including trait cognitive or somatic anxiety as a predictor of cognitive difficulties, and age as the moderator variable. Covariates included depression, stress (the Depression Anxiety Stress Scales—short form; DASS-21, Lovibond and Lovibond, 1995), gender, current mental health treatment status, and physical health status. When cognitive anxiety was the predictor variable, somatic anxiety was also included as a covariate, and vice-versa. Trait cognitive anxiety and age interacted to predict all MASQ subscales other than visual-perceptual ability. Difficulties with attention, verbal memory, and language abilities were significantly greater at higher levels of anxiety for all age groups, with the effect greatest in older adults. Difficulties with visual-spatial memory were significantly greater at higher levels of anxiety in middle-aged and older adults only. Higher trait somatic anxiety predicted difficulties with verbal memory and language ability independently of age, and interacted with age to predict language difficulties. Interestingly, age also significantly predicted less subjective difficulty with attention, independently of anxiety level. The results show that trait cognitive and somatic anxiety are both related to subjective, everyday cognitive difficulties. However, effects of trait cognitive anxiety are more robust across cognitive domains and tend to increase, or first appear, over the course of the adult lifespan.

## Introduction

The current research was aimed at investigating the potential for adult aging to moderate the effects of trait anxiety on subjective cognitive difficulties in everyday life. According to Attentional Control Theory (ACT; Eysenck et al., 2007) anxiety affects attentional processing, potentially impairing cognitive performance. Similarly, healthy adult aging has been associated with decrements in a range of cognitive processes. Despite anxiety and aging both having adverse effects on aspects of cognition, evidence surrounding the effects of anxiety on cognition in older adults remains mixed, and there is a clear need to assess the effects of anxiety on cognition across the adult lifespan.

Attentional Control Theory (ACT; Eysenck et al., 2007) is underpinned by the assumption that anxiety reduces executive control of attention (Derakshan and Eysenck, 2009). Anxiety is specifically argued to affect two attentional systems—a goal-driven system and a stimulus-driven system. Anxiety is thought to cause an imbalance between the two systems *via* increased bottom-up processing of irrelevant stimuli within the environment, thus reducing an individual’s top-down attentional control (Eysenck et al., 2007; Coombes et al., 2009). In line with ACT, high anxiety can disrupt executive functions (see Shi et al., 2019, for a meta-analysis). Specifically, anxiety affects the ability to suppress irrelevant information whilst attending to relevant information (i.e., stimulus inhibition; e.g., Ansari and Derakshan, 2010; Moser et al., 2012), to inhibit prepotent responses (e.g., Pacheco-Unguetti et al., 2012; Berggren and Derakshan, 2014), and to alternate attentional focus (i.e., shifting; e.g., Caselli et al., 2004; Ansari et al., 2008; Johnson, 2009).

Anxiety can fluctuate over time and vary in intensity. A tendency to experience anxious states frequently is defined as trait anxiety (Eysenck, 1982; Grös et al., 2007; Ree et al., 2008) and ACT emphasizes the effect of trait anxiety on cognitive performance (Eysenck et al., 2007; Eysenck and Derakshan, 2011). The dimensions of anxiety are also distinct between cognitive experiences, such as worry and apprehension, and experiences of somatic arousal, such as fast heart rate and shortness of breath (e.g., Beck et al., 1988; Ree et al., 2008). In particular, it has been assumed that anxiety impacts cognitive functions through primarily cognitive, verbal mechanisms (Eysenck et al., 2007). This is because anxiety manifests at the cognitive level as subvocalized, worried thoughts (Rapee, 1993; Wells, 1995; Hirsch and Mathews, 2012). Indeed, studies have shown that anxiety affects both verbal fluency and aspects of vocabulary (Salthouse, 2012; Gawda and Szepietowska, 2016), attentional control (Edwards et al., 2015, 2017), and working memory efficiency (Held et al., 2020; Spalding et al., 2021). However, self-reported experiences of somatic anxiety can also predict less efficient and effective cognitive performance (Schoen and Holtzer, 2017). For example, somatic anxiety has been shown adversely to affect visual working memory accuracy (Spalding et al., 2021). Few studies have investigated the distinct effects that the cognitive and somatic dimensions of anxiety may exert on cognition. It is essential to understand the distinction between these two domains, as experiences of anxiety manifest differently depending on the individual (Endler and Kocovski, 2001; Ree et al., 2008). That is, global measures of anxiety may not accurately capture the specific anxious profiles of different individuals that may contribute to adverse effects on their cognition. In the present study we therefore assessed effects of trait cognitive and somatic anxiety as separable experiences.

Regarding age-related changes in cognitive functioning, speeded, processing-intensive (i.e., “fluid”) abilities tend to decline across the adult lifespan. Notably, while age-related cognitive decline often occurs linearly across the adult lifespan, decrements tend to become more apparent after the age of 60 (Lindenberger, 2014; Schaie, 2016; Cabeza et al., 2018). In contrast, crystallized abilities, such as wisdom or vocabulary, remain relatively stable or even continue to increase (Park et al., 2002; Ardelt, 2010). Processing speed is a core ability that typically declines through the adult lifespan (Salthouse, 1996; Luo and Craik, 2008; Harada et al., 2013; Ebaid et al., 2017). As processing speed is linked to sensory, perceptual, and information processing functions, cognitive processes that are essential for moment-to-moment functioning in everyday life such as visual cognition and short-term (“working”) memory can also be impacted (Salthouse, 1996, 2019; Gregory et al., 2008; Deary et al., 2010; Brown et al., 2012; Harada et al., 2013; Guest et al., 2015). For instance, aging has been shown to affect both visual and verbal memory (Jenkins et al., 1999; Park et al., 2002; Kemps and Newson, 2006; Johnson et al., 2010; Brown et al., 2017; Swanson, 2017). Age-related decreases in visual-perceptual abilities may depend on the complexity of the cognitive task (Faubert, 2002). For example, local geometric pattern perception may be affected to a greater extent than global perception (Meng et al., 2019). This extends to visual-spatial abilities, with older adults showing specific deficits in navigation (Ariel and Moffat, 2018), which may arise from a reduced ability to process environmental landmarks (Ramanoël et al., 2020). Interestingly, aging is associated with limitations in backward, as opposed to forward, visual-spatial memory span (Brown, 2016), which may highlight important roles for processing speed and/or executive functioning. Indeed, aging, like anxiety, has been found to affect executive control of attention (Hasher et al., 1991; Madden et al., 2005; Hull et al., 2008; Harada et al., 2013; Reuter-Lorenz and Lustig, 2016).

Despite age-related increases in vocabulary size as noted above, aging can adversely affect verbal and linguistic abilities at both cognitive and perceptual levels (Wingfield and Lash, 2016). Declines in speech recognition may be due to reduced perceptual effort and/or cognitive resources required to access downstream operations for comprehension or verbal memory (Wingfield et al., 2005; Surprenant, 2007). The decline in production and recognition of words can also result in slower speech, and younger adults can outperform older adults on performance in lexical retrieval and decision making (Lima et al., 1991; Mortensen et al., 2006). Additionally, decline in language abilities might be connected to other domains such as executive function and memory, as seen in declines in verbal reasoning abilities (Harada et al., 2013; del Prado Martín, 2017) and verbal fluency (i.e., the ability to generate and search for words beginning with a certain letter, or within categories such as animals).

Importantly, while effects of anxiety and aging on cognitive performance have been observed separately, research also suggests that the impact of anxiety on cognition could be moderated by aging (Charles and Luong, 2013). The Strength and Vulnerability Integration (SAVI) model suggests that both the strengths and vulnerabilities associated with aging can influence emotion regulation across the adult lifespan (Charles and Piazza, 2009; Charles, 2010). Strengths of aging arise from the knowledge and experience acquired as individuals progress through life. As people age, there is a tendency to avoid information or situations that increase negative emotions (such as anxiety) and exert pressure on cognition (Charles and Carstensen, 2008; Charles, 2010; Charles and Luong, 2013). It has also been suggested that older adults may actively employ cognitive behavioral strategies to reappraise thoughts and behaviors more positively, and recall previous events in a more positive light (Charles and Luong, 2013). This is referred to as the “positivity effect” (Carstensen and Mikels, 2005; Ruffman et al., 2008; Knight et al., 2016). As a result, aging is typically associated with more positive emotional wellbeing overall, with older adults reporting greater happiness compared to younger adults, and emotional wellbeing “peaking” around the mid-60s–70s (Carstensen, 2006; Carstensen et al., 2011; Charles and Luong, 2013).

It has been suggested that cognitive behavioral strategies employed by older adults are consistent across a range of situations (Eldesouky and English, 2018). However, as proposed by the SAVI model, these consistent approaches to situational judgment can potentially impair older adults’ ability to cope with highly aversive situations (Charles and Piazza, 2009; Charles, 2010). The theory of cognitive control argues that older adults require cognitive processing resources to successfully regulate their emotions, and the effects of anxiety also pose demands on the availability of these cognitive resources (Charles and Luong, 2013). For example, unavoidable, distressing situations such as bereavement, social isolation and illness can increase feelings of anxiety and pose a greater threat to more vulnerable cognitive processing and physiological systems (Charles and Carstensen, 2010; Charles and Luong, 2013). This could potentially indicate that the decline seen in older adults is due to allocation of resources, that they might prioritize emotion over knowledge, or that with less cognitive control they might have difficulty implementing emotion regulation strategies (Mather and Carstensen, 2005), for example due to a poorer threshold for executive functioning (Scheibe and Blanchard-Fields, 2009). The literature supports the co-occurrence of anxiety and cognitive decline in later life; however, the direction of this relationship is unclear regarding whether the declines in cognitive performance subsequently occur as a result of greater anxiety, or if increased anxiety is due to reduced cognitive performance (Petkus et al., 2017).

The few studies that have examined the role of anxiety in older adults’ cognitive performance have thus far focused on attentional processing and verbal memory. Furthermore, few studies have considered the separate influences that cognitive and somatic anxiety may exert on older adults’ cognition. Mella et al. (2020) found that trait cognitive anxiety predicted poorer cognitive flexibility and processing speed in a sample of older adults. Beaudoin (2018) similarly found a mediating effect of worry (i.e., cognitive anxiety), but not emotionality (i.e., somatic anxiety), on the relationship between memory self-efficacy and processing efficiency in a verbal episodic memory task. Results have, however, proven inconsistent with respect to the relative effects of cognitive and somatic anxiety on cognition. Perhaps counterintuitively, Schoen and Holtzer (2017) found that somatic anxiety, but not cognitive anxiety, was associated with reduced attentional performance in measures of executive attention that rely on processing speed. It is therefore necessary to further clarify the effects of these anxiety dimensions on older adults’ cognition across domains. It is also pertinent to address these relationships across the adult lifespan rather than simply within young or older age, with the aim of achieving a clearer understanding of the relationships amongst aging, trait cognitive and somatic anxiety, and cognition.

The aim of the present study was therefore to assess the effect of anxiety on subjective cognitive abilities, while also accounting for the potential moderating effect of healthy adult aging. Although an indirect measure of cognitive ability, self-reported cognitive difficulties allow for the assessment of perceived errors/difficulty in everyday cognition across a variety of key domains (e.g., attentional control and visual/verbal abilities). This approach should therefore be useful for understanding cognitive functioning in the context of relatively stable, dispositional (i.e., trait) anxiety as well as healthy aging. Furthermore, there is important theoretical value in further studying the effects of aging on subjective cognition. While objective cognitive difficulties are consistently observed in older adults, subjective difficulties are less consistently observed (Carrigan and Barkus, 2016). It has been suggested that the failure to detect an association between age and self-reported cognitive failures is due to older adults comparing themselves to their peers, instead of their own cognitive functioning in youth. Thus, older adults might report better subjective functioning than they would exhibit objectively (Newson and Kemps, 2008). By comparison, anxiety has been associated with self-perceived everyday cognitive failures (Broadbent et al., 1982; Mahoney et al., 1998; Mecacci et al., 2004; Righi et al., 2009). Older adults increasingly demonstrate anxiety around age-related cognitive decline, a phenomenon referred to as “dementia worry” (Kessler et al., 2012). It is therefore possible that subjective cognitive failures may manifest in aging to a greater extent if self-reported levels of anxiety—particularly anxious thoughts—are higher. Furthermore, subjective cognitive difficulties may be a marker for future cognitive impairment and dementia (Reisberg et al., 2010; Opdebeeck et al., 2019). High levels of anxiety and anxiety disorders in older adults are indeed associated with impairment in functionality and cognitive decline as a long-term consequence (Lenze and Wetherell, 2009; Charles and Luong, 2013; Knight et al., 2016). Adopting subjective measures of cognition in the present study, then, can help to illuminate the extent to which anxiety in older adults is related to their subjective everyday cognitive functioning, and potentially future cognitive decline.

We predicted that trait anxiety, and especially cognitive as compared with somatic experiences of anxiety, would be associated with subjective everyday cognitive functioning. We also predicted that attention, verbal memory, and language abilities would be more sensitive to trait cognitive anxiety levels than visual-spatial and visual-perceptual abilities (e.g., Vytal et al., 2013), in line with the assumptions within ACT (Eysenck et al., 2007), and recent studies examining the impact of anxiety on older adults’ cognitive performance (Schoen and Holtzer, 2017; Beaudoin, 2018; Mella et al., 2020). Importantly, however, we predicted that age would moderate the relationship between trait anxiety and cognition. Higher trait anxiety reflects more frequent experiences of anxious states in response to stressors (Eysenck et al., 2007), and while older adults tend to report being happier, effects of unavoidable aversive moods and situations may impact older adults to a greater extent than they do younger adults (Charles, 2010). We therefore expected that higher anxiety would be related to greater subjective everyday cognitive difficulties, especially in older adults.

## Materials and Methods

### Participants

Prior to commencement, the study received ethical approval from the School of Psychological Sciences and Health Ethics Committee at the University of Strathclyde. Participants were 286 adults (68 male, 218 female) aged 18–93 (*M* = 42.47, *SD* = 20.82) recruited through an undergraduate participant pool and older adult participant panels at the University of Strathclyde, and by advertising through social media and personal acquaintances. All participants resided in the United Kingdom and self-reported no diagnosed cognitive impairments or neurological disorders. A power analysis for a linear regression with three predictor variables (the predictor, moderator, and their interaction term) and six control variables, using G^∗^Power 3.1 (Faul et al., 2009), provided an estimated sample size of 119, based on high power to detect a medium effect size (*f*^2^ = 0.15; α = 0.05; power = 0.95).

Further information on the overall characteristics of the participant sample may be viewed in Table 1. However, it is also useful to provide an indication of mental health treatment status and general health status by age group. Regarding mental health treatment, of those participants typically considered “younger” adults (aged 18–35 years), 26 participants (19.8%) were currently receiving mental health treatment while 103 participants (78.6%) were not receiving treatment and two participants (1.5%) preferred not to indicate. In middle-aged adults (aged 36–59 years), 18 participants (23.4%) were currently receiving mental health treatment, 58 participants (75.3%) were not receiving treatment, and one participant (1.3%) preferred not to indicate. In older adults (aged 60–93 years), one participant (1.3%) was currently receiving mental health treatment, 76 participants (97.4%) were not receiving treatment, and one participant preferred not to indicate. In terms of general health, 28 (21.4%) younger adults, reported having very poor-to-fair general health, and 103 (78.6%) reported having quite good or very good health. In middle-aged adults, 25 participants (23.4%) reported having quite poor or fair health, and 52 participants (76.6%) reported having quite good or very good health. In older adults, 12 participants (15.4%) reported having very poor-to-fair health, and 66 participants (84.6%) reported having quite good or very good health. As the proportion of participants currently receiving mental health treatment and experiencing poorer general health varied somewhat across the age groups, we controlled for these variables in our analyses.

**TABLE 1:** Participants’ socio-demographic data.

Variables	*n* (%)
**Age (*M* = 42.47; *SD* = 20.82)**	
Young (18–35 years)	131(45.8%)
Middle-aged (36–59 years)	77(26.9%)
Older (60–93 years)	78(27.3%)
**Gender**	
Female	218(76.2%)
Male	68(23.8%)
**Ethnicity**	
White	278(97.2%)
Asian, Asian Scottish/British	4(1.4%)
African, African Scottish/British	1(0.3%)
Mixed/Multiple	2(0.7%)
Other (“Scottish”)	1(0.3%)
**English first language?**	
Yes	281(98.3%)
No	5(1.7%)
**Education**	
No schooling completed	1(0.4%)
High School	71(24.9%)
Further education/college	85(29.8%)
University undergraduate	64(22.5%)
Postgraduate	59(20.7%)
Doctorate	4(1.4%)
Prefer not to say	1(0.4%)
**Employment status**	
Full-time employment	72(25.4%)
Part-time employment	56(19.7%)
Unemployed	15(5.3%)
Self-employment	8(2.8%)
Home-maker	4(1.4%)
Student	58(20.4%)
Retired	70(24.6%)
Prefer not to say	1(0.4%)
**General health**	
Very poor	6(2.1%)
Quite poor	13(4.5%)
Fair	46(16.1%)
Quite good	130(45.5%)
Very good	91(31.8%)
**Ever had mental health diagnosis**	
Yes	70(24.5%)
No	208(72.7%)
Prefer not to say	8(2.8%)
**Currently receiving mental health treatment**	
Yes	45(15.7%)
No	237(82.9%)
Prefer not to say	4(1.4%)

*NB: % calculations exclude missing data.*

### Design

A cross-sectional survey design was used. In each moderated regression analysis, the predictor variables were either trait cognitive or somatic anxiety, and age was included as a moderator variable. Gender, depression, stress, current mental health treatment status, and general health status were included as covariates, as was either anxiety dimension (trait cognitive or somatic anxiety) when the other anxiety dimension was included as a predictor. The outcome variables were subjective difficulty with language, attention/concentration, verbal memory, visual-spatial memory, and visual-perceptual ability.

### Materials

#### Anxiety Measure

The State-Trait Inventory for Cognitive and Somatic Anxiety (STICSA; Ree et al., 2008) is a 21-item self-report scale discriminating between cognitive (10 items), and somatic symptoms (11 items) of both state and trait anxiety (Grös et al., 2007). Participants responded to items on a 4-point Likert scale ranging from “almost never” to “almost always” in response to statements such as “I think others won’t approve of me” (cognitive) and “my heart beats fast” (somatic). Higher scores indicate higher levels of anxiety for both cognitive anxiety (ranging from 10 to 40) and somatic anxiety (ranging from 11 to 44). The STICSA scales have shown to be reliable in clinical and non-clinical samples (internal consistency > 0.90; Grös et al., 2007). It has also been found as a reliable and valid measure in older populations (Balsamo et al., 2015).

#### Measure of Cognitive Difficulties

The 38-item Multiple Ability Self-Report Questionnaire (MASQ; Seidenberg et al., 1994) was used to measure subjective cognitive difficulties in everyday life across five subscales: language (e.g., “When talking, I have difficulty conveying precisely what I mean”); attention/concentration (e.g., “I ask people to repeat themselves because my mind wanders during conversations”); verbal memory (e.g., “I forget to mention important issues during conversations”); visual-spatial memory (e.g., “I have difficulty finding stores in a mall even if I have been there before”); and visual-perceptual ability (e.g., “I have difficulty locating a friend in a crowd of people”). The first four subscales each include eight items measured on a 5-point Likert scale from 1 (“never”) to 5 (“always”; minimum score per subscale = 8, maximum = 40). The last subscale (visual-perceptual ability) includes six items measured on the same 5-point Likert scale (minimum score = 6, maximum = 30). Across all subscales, higher scores indicate greater cognitive difficulty. The MASQ has been shown to be reliable (Cronbach’s α = 0.92; > 0.70 for all subscales; Seidenberg et al., 1994) and has been used across a variety of psychological and clinical contexts, including with older adults (e.g., Judges et al., 2017; McDonald et al., 2017; Nicholls et al., 2021).

#### Depression and Stress Measures

The Depression Anxiety Stress Scales (DASS-21; Lovibond and Lovibond, 1995) were administered in order to account for depression and stress as covariates. The DASS-21 comprises 21 items across three subscales which assess dysphoric mood (i.e., depression), fear symptoms and autonomic arousal (i.e., anxiety) and tension and agitation symptoms (i.e., stress). Participants respond to various statements indicating their feelings over the past week (e.g., “I couldn’t seem to experience any positive feeling at all”—depression; “I felt I was close to panic”—anxiety; “I found it difficult to relax”—stress). Responses were given on a 4-point Likert scale ranging from 0 (“did not apply to me at all”) to 3 (“applied to me very much, or most of the time”). Raw scores are multiplied by two to calculate the total score for each subscale based on the full DASS 42-item scale. Higher scores indicate higher levels of depression, anxiety and stress.

### Procedure

The survey was administered online *via* Qualtrics during February 2021. Informed consent was first obtained from all participants, and then participants were asked to confirm that they met the inclusion criteria. The survey then comprised initial demographic questions followed by the STICSA Trait then State subscales (Ree et al., 2008), the DASS-21 (Lovibond and Lovibond, 1995), and finally the MASQ (Seidenberg et al., 1994). The survey took approximately 20 min to complete, but participants had the opportunity to pause and finish it up to 48 h after starting, providing they used the same device. Participants were debriefed following completion of the survey. Responses to individual items were reverse-scored where required before calculating participants’ scores on each sub-scale.

### Data Analysis

A series of moderated regression analyses were conducted using the Process v3 macro for SPSS (Hayes, 2018). Trait cognitive and somatic anxiety were treated, in turn, as predictors of each of the subjective cognitive abilities, with participant age included as a moderator. Gender, depression, stress, current mental health treatment status (currently undergoing treatment, or not undergoing treatment) and general health status (very poor-to-fair, or quite good-to-very good) were also included as covariates in the analyses. For analyses in which trait cognitive anxiety was the predictor variable, trait somatic anxiety was also included as a covariate, and vice-versa.

Simple slopes were used to further explore any significant interactions, and were produced using set mean-centered anxiety and age values derived from the Process analyses. Following Hayes (2018), “low anxiety” and “younger adults” were represented by scores at the 16th percentile of their distribution, “moderate anxiety” and “middle-aged adults” were represented by scores at the 50th percentile, and “high anxiety” and “older adults” were represented by scores at the 84th percentile. Note, these values are indicative of relatively low, moderate, and high scores on each variable specifically within the present study sample. The chosen percentiles represent one standard deviation below the mean, the mean, and one standard deviation above the mean, respectively, if the moderator is normally distributed, and ensure that scores representing low and high levels are always within the range of the observed data regardless of distribution (Hayes, 2018).

## Results

Correlations amongst the predictor, control, and outcome variables can be viewed in Table 2. Age, trait cognitive anxiety, and trait somatic anxiety were all significantly associated with subjective difficulty across all cognitive domains measured by the MASQ (Seidenberg et al., 1994). Higher trait cognitive and somatic anxiety were associated with greater cognitive difficulty. However, age was negatively associated with all of the emotion and cognitive measures, indicating less subjective difficulty with both cognition and emotion across the lifespan overall.

**TABLE 2:** Mean values (with *SD*s) and Pearson correlations amongst predictor, control, and outcome variables.

	*M* (*SD*)	1	2	3	4	5	6	7	8	9	10	11	12
1. Age	42.47 (20.82)	−											
2. Gender		–0.06	−										
3. Receiving mental health treatment		0.15*	0.08	−									
4. General health status		0.02	–0.04	0.22***	−								
5. Depression (DASS-21)	26.18 (10.39)	−0.37***	−0.13*	−0.22***	−0.26***	−							
6. Stress (DASS-21)	27.36 (9.65)	−0.42***	−0.17**	−0.25***	−0.22***	0.76***	−						
7. Trait cognitive anxiety (STICSA)	20.97 (7.47)	−0.43***	–0.10	−0.35***	−0.27***	0.73***	0.78***	−					
8. Trait somatic anxiety (STICSA)	18.08 (5.79)	−0.34***	−0.15*	−0.36***	−0.37***	0.63***	0.70***	0.74***	−				
9. MASQ Attention	19.46 (5.57)	−0.49***	–0.03	−0.26***	−0.20**	0.52***	0.56***	0.65***	0.51***	−			
10. MASQ Verbal memory	19.05 (5.92)	−0.30***	–0.06	−0.25***	−0.18**	0.48***	0.47***	0.57***	0.49***	0.76***	−		
11. MASQ Language	17.06 (5.23)	−0.28***	–0.10	−0.23***	−0.18**	0.58***	0.59***	0.64***	0.57***	0.67***	0.69***	−	
12. MASQ Visual-perceptual ability	12.89 (4.56)	−0.14*	−0.18**	−0.22***	−0.18**	0.40***	0.39***	0.41***	0.40***	0.58***	0.55***	0.56***	−
13. MASQ Visual-spatial memory	16.63 (5.11)	−0.25***	–0.08	−0.21***	−0.19**	0.37***	0.40***	0.43***	0.37***	0.71***	0.71***	0.61***	0.69***

NB: *< 0.05; **< 0.01; and ***< 0.001.

*Overall *N* = 286, however, five respondents did not complete MASQ language, visual-perceptual ability, and visual-spatial memory scales; six participants did not complete MASQ attention scale, seven participants did not complete STICSA subscales; eight participants did not complete DASS subscales; 10 participants did not complete MASQ verbal memory scale.*

### Trait Cognitive Anxiety

Summarizing first the models including trait cognitive anxiety as a predictor, the model predicting attention was significant, *F*(9, 244) = 26.18, *p* < 0.001, and predicted 49% of the variance (*R* = 0.70, *R*^2^ = 0.49, *MSE* = 16.30). The model predicting verbal memory was significant, *F*(9, 240) = 15.74, *p* < 0.001, and predicted 37% of the variance (*R* = 0.61, *R*^2^ = 0.37, *MSE* = 22.67). The model predicting language was significant, *F*(9, 244) = 23.60, *p* < 0.001, and predicted 47% of the variance (*R* = 0.68, *R*^2^ = 0.47, *MSE* = 15.13). The model predicting visual-perceptual ability was also significant, *F*(9, 244) = 8.67, *p* < 0.001, and predicted 24% of the variance (*R* = 0.49, *R*^2^ = 0.24, *MSE* = 16.80). Finally, the model predicting visual-spatial memory was significant, *F*(9, 243) = 7.64, *p* < 0.001, and predicted 22% of the variance (*R* = 0.47, *R*^2^ = 0.22, *MSE* = 20.89).

Parameter estimates and tests of significance for moderation analyses involving trait cognitive anxiety can be viewed in Table 3. The interaction between trait cognitive anxiety and age significantly predicted subjective difficulties across most of the cognitive domains, which are now explored in turn.

**TABLE 3:** Unstandardized coefficients, significance tests, and 95% confidence intervals for predictors of subjective cognitive difficulties in each moderated regression analysis including trait cognitive anxiety as a predictor.

	*N*	Unstandardized coefficients				95% confidence intervals for B	
		*B*	*SE*	*t*	*p*	Lower bound	Upper bound
**Attention**	254						
Trait cognitive anxiety		0.32	0.07	4.90	< 0.001	0.19	0.45
Age		–0.06	0.01	–4.30	< 0.001	–0.09	–0.03
Trait cognitive anxiety × age		0.005	0.002	2.50	0.013	0.001	0.01
**Visual-perceptual ability**	254						
Trait cognitive anxiety		0.11	0.07	1.67	0.096	–0.02	0.24
Age		0.01	0.01	0.90	0.369	–0.02	0.04
Trait cognitive anxiety × age		0.004	0.002	1.76	0.080	–0.000	0.01
**Visual-spatial memory**	253						
Trait cognitive anxiety		0.19	0.08	2.57	0.011	0.04	0.34
Age		–0.01	0.02	–0.73	0.468	–0.04	0.02
Trait cognitive anxiety × age		0.005	0.002	2.06	0.040	0.000	0.01
**Verbal memory**	250						
Trait cognitive anxiety		0.33	0.08	4.14	< 0.001	0.17	0.49
Age		–0.01	0.02	–0.50	0.618	–0.04	0.03
Trait cognitive anxiety × age		0.01	0.002	2.42	0.016	0.001	0.01
**Language**	254						
Trait cognitive anxiety		0.29	0.06	4.62	< 0.001	0.17	0.42
Age		0.02	0.01	1.23	0.219	–0.01	0.04
Trait cognitive anxiety × age		0.006	0.002	2.84	0.005	0.002	0.01

**FIGURE 1:**
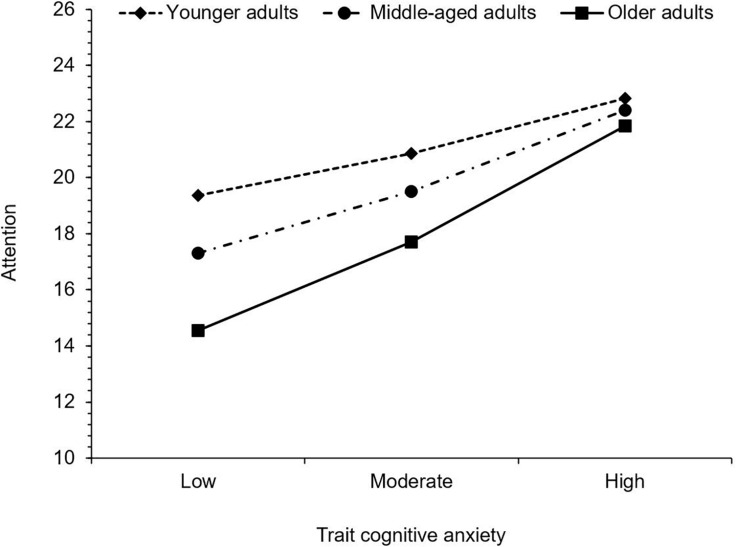
Simple slopes illustrating the significant interaction between trait cognitive anxiety and age on attention. Higher values indicate greater levels of subjective cognitive difficulty.

First, regarding attention, trait cognitive anxiety was positively associated with MASQ scores. As trait cognitive anxiety increased, so too did subjective difficulty with attention. Age was negatively associated with MASQ scores, indicating that with increasing age, subjective difficulty with attention decreased. Furthermore, the interaction between trait cognitive anxiety and age was also significant. Trait cognitive anxiety was significantly associated with attention difficulty in young adults, *b* = 0.21, 95% CI (0.08, 0.35), *t* = 3.04, *p* = 0.003, middle-aged adults, *b* = 0.31, 95% CI (0.19, 0.44), *t* = 4.82, *p* < 0.001, and older adults, *b* = 0.45, 95% CI (0.27, 0.63), *t* = 4.88, *p* < 0.001, with the effect size increasing progressively with age (see Figure 1).

**FIGURE 2:**
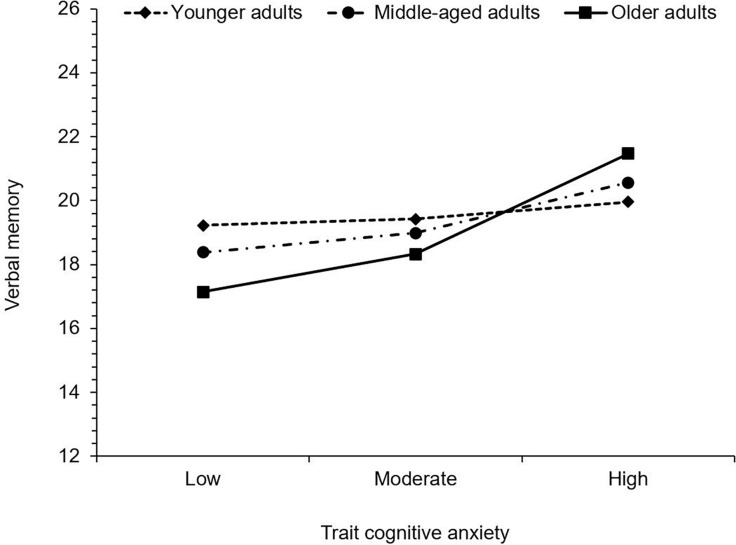
Simple slopes illustrating the significant interactions between trait cognitive anxiety and age on verbal memory. Higher values indicate greater levels of subjective cognitive difficulty.

Regarding verbal memory, trait cognitive anxiety was positively associated with MASQ scores. Thus, as trait cognitive anxiety increased, so too did subjective difficulty with verbal memory. Age was not significantly associated with verbal memory, however, the interaction between trait cognitive anxiety and age was significant. Trait cognitive anxiety was associated with greater difficulty in young adults, *b* = 0.21, 95% CI (0.04, 0.37), *t* = 2.43, *p* = 0.016, middle-aged adults, *b* = 0.32, 95% CI (0.16, 0.47), *t* = 4.02, *p* < 0.001, and older adults, *b* = 0.48, 95% CI (0.26, 0.70), *t* = 4.31, *p* < 0.001, again with the effect size increasing with age (see Figure 2).

**FIGURE 3:**
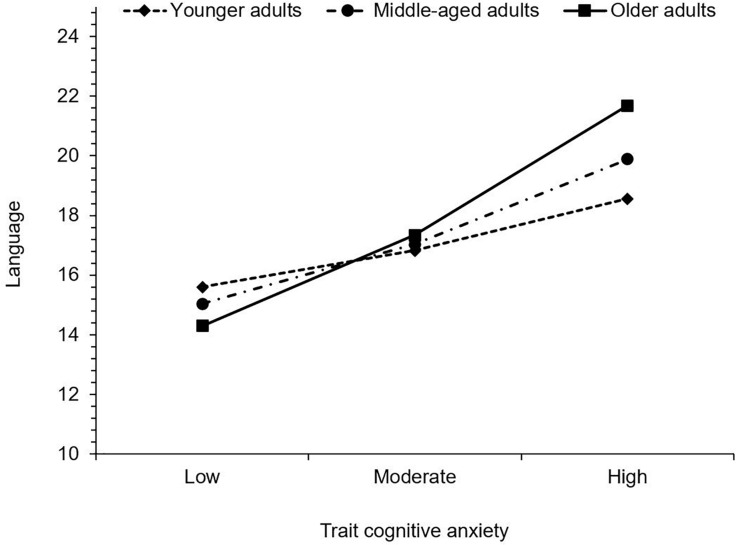
Simple slopes illustrating the significant interaction between trait cognitive anxiety and age on language. Higher values indicate greater levels of subjective cognitive difficulty.

Considering language, trait cognitive anxiety was also positively associated with MASQ scores, such that as trait cognitive anxiety increased, so too did subjective difficulty with language. Age was not significantly associated with language. The interaction between trait cognitive anxiety and age was, however, significant. Trait cognitive anxiety was associated with greater difficulty with language in young adults, *b* = 0.17, 95% CI (0.04, 0.31), *t* = 2.57, *p* = 0.011, middle-aged adults, *b* = 0.29, 95% CI (0.16, 0.41), *t* = 4.53, *p* < 0.001, and older adults, *b* = 0.44, 95% CI (0.26, 0.61), *t* = 4.88, *p* < 0.001, and again the effect size increased with age (see Figure 3).

In summary, then, greater difficulties with attention, verbal memory, and language abilities were reported at higher levels of anxiety in all age groups, but the effect was stronger with increasing age.

Regarding visual-perceptual ability, neither trait cognitive anxiety nor age was significantly associated with MASQ scores. The interaction was also not significant. Only the gender control variable was significantly associated with visual-perceptual ability, *b* = −1.26, 95% CI (−2.49, −0.03), *t* = −2.01, *p* = 0.046, with females reporting greater difficulty with visual-perceptual abilities than males.

**FIGURE 4:**
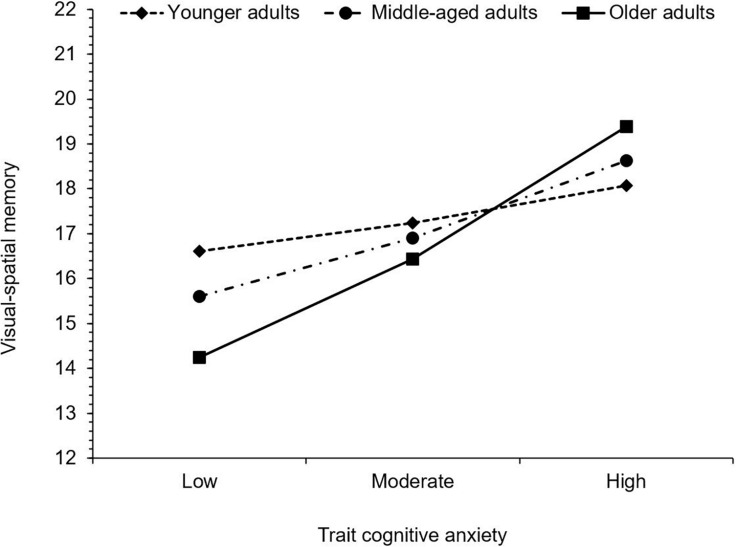
Simple slopes illustrating the significant interaction between trait cognitive anxiety and age on visual*−*spatial memory. Higher values indicate greater levels of subjective cognitive difficulty.

Finally, for visual-spatial memory, trait cognitive anxiety was positively associated with MASQ scores, thus as trait cognitive anxiety increased, so too did subjective difficulties with visual-spatial memory. Age was not significantly associated with visual-spatial memory. The interaction between trait cognitive anxiety and age was, however, significant. Trait cognitive anxiety was not significantly associated with visual-spatial memory in younger adults, *b* = 0.09, 95% CI (−0.07, 0.25), *t* = 1.13, *p* = 0.261, but there was a significant positive association between trait cognitive anxiety and difficulty with visual-spatial memory in middle-aged adults, *b* = 0.19, 95% CI (0.04, 0.33), *t* = 2.49, *p* = 0.014, and older adults *b* = 0.31, 95% CI (0.11, 0.52), *t* = 2.97, *p* = 0.003. Thus, greater subjective difficulties with visual-spatial memory was related to higher levels of anxiety in middle-aged and older adults, but not in younger adults (see Figure 4).

### Trait Somatic Anxiety

Summarizing each model involving trait somatic anxiety, the model predicting attention was significant, *F*(9, 244) = 25.08, *p* < 0.001, and predicted 48% of the variance (*R* = 0.69, *R*^2^ = 0.48, *MSE* = 16.65). The model predicting verbal memory was significant, *F*(9, 240) = 15.60, *p* < 0.001, and predicted 37% of the variance (*R* = 0.61, *R*^2^ = 0.37, *MSE* = 22.74). The model predicting language was significant, *F*(9, 244) = 22.50, *p* < 0.001, and predicted 45% of the variance (*R* = 0.67, *R*^2^ = 0.45, *MSE* = 15.47). The model predicting visual-perceptual ability was also significant, *F*(9, 244) = 8.50, *p* < 0.001, and predicted 24% of the variance (*R* = 0.49, *R*^2^ = 0.24, *MSE* = 16.88). Finally, the model predicting visual-spatial memory was significant, *F*(9, 243) = 7.22, *p* < 0.001, and predicted 21% of the variance (*R* = 0.46, *R*^2^ = 0.21, *MSE* = 21.14). Parameter estimates and tests of significance for moderation analyses involving trait somatic anxiety can be viewed in Table 4.

**TABLE 4:** Unstandardized coefficients, significance tests, and 95% confidence intervals for predictors of subjective cognitive difficulties in each moderated regression analysis including trait somatic anxiety as a predictor.

	*N*	Unstandardized coefficients				95% confidence intervals for B	
		*B*	*SE*	*t*	*p*	Lower bound	Upper bound
**Attention**	254						
Trait somatic anxiety		0.000	0.08	0.00	0.996	–0.15	0.15
Age		–0.07	0.01	–4.60	< 0.001	–0.10	–0.04
Trait somatic anxiety × age		0.003	0.003	1.06	0.291	–0.002	0.01
**Visual-perceptual ability**	254						
Trait somatic anxiety		0.09	0.08	1.23	0.222	–0.06	0.25
Age		0.01	0.01	0.84	0.403	–0.02	0.04
Trait somatic anxiety × age		0.004	0.003	1.39	0.166	–0.002	0.01
**Visual-spatial memory**	253						
Trait somatic anxiety		0.04	0.09	0.49	0.624	–0.13	0.21
Age		–0.02	0.02	–0.95	0.341	–0.05	0.02
Trait somatic anxiety × age		0.003	0.003	1.11	0.267	–0.003	0.01
**Verbal memory**	250						
Trait somatic anxiety		0.22	0.09	2.42	0.017	0.04	0.39
Age		–0.01	0.02	–0.49	0.626	–0.04	0.03
Trait somatic anxiety × age		0.007	0.003	2.25	0.025	0.001	0.01
**Language**	254						
Trait somatic anxiety		0.19	0.07	2.60	0.010	0.05	0.33
Age		0.01	0.01	0.92	0.361	–0.02	0.04
Trait somatic anxiety × age		0.004	0.003	1.62	0.107	–0.001	0.01

Regarding attention, trait somatic anxiety was not significantly associated with MASQ scores. However, age was negatively associated with attention, therefore as age increased, subjective difficulties with attention decreased. The interaction between trait somatic anxiety and age was not significant.

**FIGURE 5:**
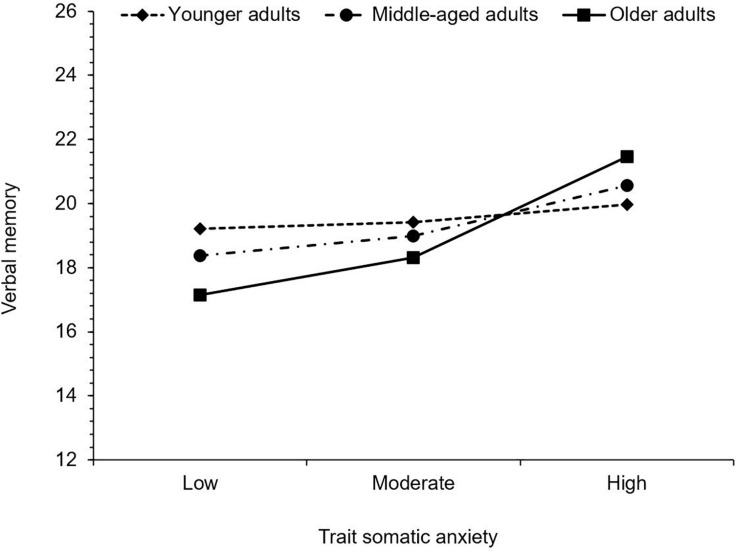
Simple slopes illustrating the significant interaction between trait somatic anxiety and age on verbal memory. Higher values indicate greater levels of subjective cognitive difficulty.

For verbal memory, trait somatic anxiety was positively associated with MASQ scores, therefore as trait somatic anxiety increased, subjective difficulty with verbal memory also increased. Age was not significantly associated with verbal memory. The interaction between trait somatic anxiety and age was, however, significant. Trait somatic anxiety was not significantly associated with verbal memory in younger adults, *b* = 0.07, 95% CI (−0.12, 0.25), *t* = 0.72, *p* = 0.473, but there was a significant positive association between trait somatic anxiety and verbal memory difficulty in middle-aged adults, *b* = 0.20, 95% CI (0.03, 0.37), *t* = 2.28, *p* = 0.023, and older adults *b* = 0.39, 95% CI (0.12, 0.66), *t* = 2.88, *p* = 0.004. Thus, greater subjective difficulties with verbal memory were reported at higher levels of trait somatic anxiety in middle-aged and older adults, but not in younger adults (see Figure 5).

Regarding language, trait somatic anxiety was positively associated with MASQ scores, therefore as trait somatic anxiety increased, subjective language difficulties also increased. Age was not significantly associated with language difficulties, and neither was the interaction.

Considering visual-perceptual ability, trait somatic anxiety was not significantly associated with MASQ scores, and neither was age and the interaction. Only the gender covariate was significantly associated with visual-perceptual ability, *b* = −1.30, 95% CI (−2.54, −0.06), *t* = −2.06, *p* = 0.040. As with the analysis involving trait cognitive anxiety as the predictor of visual-perceptual ability, females reported greater visual-perceptual difficulty than did males.

Finally, for visual-spatial memory, trait somatic anxiety was not significantly associated with MASQ scores, and neither was age and the interaction. Only the trait cognitive anxiety covariate was significantly and positively associated with visual-spatial memory, *b* = 0.16, 95% CI (0.01, 0.30), *t* = 2.11, *p* = 0.036, indicating greater difficulty with increased trait cognitive anxiety.

## Discussion

In the present research we investigated the effect of trait anxiety dimensions on self-reported cognitive difficulties in everyday life, and whether these relationships were moderated by adult age. Age moderated the relationship between trait cognitive anxiety and most of the measures of subjective cognitive difficulties. For attention, verbal memory, and language, trait cognitive anxiety predicted greater difficulty in all age groups, however, the effect increased with age. Regarding attention specifically, age was also independently associated with less cognitive difficulty. Regarding visual-spatial memory, trait cognitive anxiety independently predicted greater cognitive difficulty, but also interacted with age. Here, younger adults did not experience greater cognitive difficulty at higher levels of anxiety, but middle-aged and older adults did so. There were no associations between trait cognitive anxiety or age and visual-perceptual ability. By comparison, in the analyses that included trait somatic anxiety as the anxiety predictor variable, this independently predicted reduced verbal memory and language abilities, while age again predicted less difficulty with attention. Trait somatic anxiety also interacted with age only to predict verbal memory. Here, difficulties did not increase with anxiety in younger adults, but did so in middle-aged and older adults. Overall then, from middle age, adults appear more consistently vulnerable to the effects of higher trait cognitive anxiety, as opposed to trait somatic anxiety, on subjective cognitive difficulties, specifically regarding attention, verbal memory, language, and visual-spatial memory, but not visual-perceptual ability.

### Age, Trait Cognitive Anxiety, and Subjective Cognitive Difficulties

The findings suggest that age moderates the effect of trait cognitive anxiety and trait somatic anxiety on aspects of everyday subjective cognition. This is consistent with the SAVI model (Charles, 2010), which posits that there are limitations of the positivity effect of aging on wellbeing, and which otherwise predicts greater emotional regulation and wellbeing across the adult lifespan. Specifically, it is suggested that with unavoidable or chronic stressors and higher levels of negative mood such as worry, older adults may demonstrate worse cognitive performance than younger adults (Charles, 2010; Knight et al., 2016).

In the present study it was observed that subjective cognitive difficulties tended to be greater for middle-aged and older adults with higher levels of trait cognitive anxiety. It is suggested in SAVI (Charles, 2010) that, at high levels of anxiety, pressure is exerted on cognitive processing systems. Anxiety, in combination with the consistent use of resource-intensive cognitive strategies intended to reappraise information or situations more positively or avoid them, may result in older adults’ cognitive performance being impaired (Charles and Luong, 2013). It has previously been observed that higher levels of trait anxiety have the ability to impair memory suppression, for example (Mariz et al., 2014). Thus, it is suggested that aging may cause increased difficulty regulating emotions under certain conditions, and therefore the influence of anxious thoughts may become more apparent.

The present results confirm that trait cognitive anxiety has a specific role in the relationship between aging and attentional processes. This is consistent with another recent study examining the impacts of cognitive and somatic anxiety on older adults’ processing speed, cognitive flexibility, and working memory (Mella et al., 2020). The specific role of cognitive anxiety may be due to demands placed on resources in working memory which can regulate the effect of anxiety on cognition in older adults, who may have more limited working memory/executive attentional resources (Mella et al., 2020). This is also consistent with suggestions that the efficiency and reliability of different neurocognitive components varies with age (Lustig and Lin, 2016), and the theory of cognitive control (Mather and Carstensen, 2005; Charles and Luong, 2013). This can be seen within the present middle-aged and older adult groups, where rising levels of trait cognitive anxiety became disruptive, or more disruptive, to aspects of subjective cognition from middle age. Thus, with aging, cognitive difficulties tended to appear or increase when combined with higher trait cognitive anxiety. Consistent with the theory of cognitive control, aging may affect emotion-regulating processes involving attention and working memory (e.g., Mather and Carstensen, 2005; Scheibe and Blanchard-Fields, 2009). As cognitive control and emotion regulation domains are connected, this may mean that, due to older adults’ more limited resources to regulate worrying thoughts (trait cognitive anxiety), cognitive performance is affected (Beaudreau and O’Hara, 2008).

Another important outcome is that the present results provide some initial explanation as to why subjective cognitive difficulties have not been consistently reported to a greater degree in older adults as compared to younger adults (see Carrigan and Barkus, 2016). To return again to SAVI (Charles, 2010; Charles and Luong, 2013), it is suggested that older adults may see their cognitive performance decline when strategies intended to improve mood are utilized during aversive situations or negative mood. That is, subjective cognition may not be adversely affected in older adults who are not, for example, highly anxious. However, when older adults do experience more anxiety, subjective appraisal of their cognitive difficulties may increase. If older adults are more worried about their cognitive ability (e.g., Kessler et al., 2012), their cognitive resources may be focused upon these anxious cognitions, and/or attempts to alleviate the anxiety, rather than on emotional regulation strategies or behaviors. Therefore, older adults may subjectively rate their cognitive abilities as being worse when they are anxious, or the combination of worry and the use of taxing cognitive strategies may actually impact their overall cognitive performance. The present data present a pattern consistent with this view that warrants further exploration. Depending on the specific cognitive domain, trait cognitive anxiety either affected only middle-aged or older adults, or affected these groups to a greater extent than younger adults. Referring to the patterns observed in the simple slopes analyses, the descriptive data indicate that older adults generally reported less cognitive difficulty at low levels of anxiety as compared with the other age groups, particularly young adults. This was also specifically observed in the significant negative association overall between age and attention difficulties, indicating less attention difficulties with age. In line with the significant increase in cognitive difficulty as a product of increased anxiety and older age, the results present promising support for SAVI (Charles, 2010; Charles and Luong, 2013) as an account of the relationship between aging, emotion, and cognition. Older adults reported a steeper increase in cognitive difficulty as anxiety levels increased, which is to be expected if their cognitive resources were taxed to a greater extent than younger adults. In parallel, though, the positivity effect can also potentially explain why older adults appear to report less cognitive difficulty at lower levels of anxiety, and, where a significant main effect of age was observed, age was negatively associated with cognitive difficulties. If older adults are more likely to employ strategies such as positively appraising thoughts and behaviors (Ruffman et al., 2008; Knight et al., 2016), it would follow that they would report less subjective difficulty with everyday cognition. However, further inferential analyses of potential between-groups differences in cognitive difficulties across age groups is required to determine whether older adults indeed experience, subjectively, significantly less cognitive difficulty at lower levels of anxiety.

The results have an important practical impact in this context. While the results suggest that cognitive anxiety affects cognitive functioning to a greater extent as adults grow older, the inverse of this is that older adults’ cognitive abilities may benefit if they are able to maintain lower levels of anxiety. Particularly as subjective cognitive difficulties may be associated with future cognitive decline and dementia (Reisberg et al., 2010; Opdebeeck et al., 2019), there is an incentive to reduce levels of anxiety in older adults, not only to improve their mood, but also to potentially improve their cognitive functioning over the longer term as well. Of course, this possibility is suggested only tentatively, given that it is still necessary to first establish a causal relationship between anxiety and subjective cognitive difficulty across the adult lifespan, and to determine the direction of the relationship.

### Trait Anxiety and Cognitive Difficulty

Outside of the moderating effects of aging, the findings suggest more generally that with higher levels of trait cognitive and somatic anxiety, there will be higher levels of subjective cognitive difficulty. This is consistent with Attentional Control Theory (ACT; Eysenck et al., 2007; see also Eysenck and Derakshan, 2011; Berggren and Derakshan, 2013), which proposes that highly anxious individuals are less efficient, and sometimes less effective, in the performance of cognitive tasks. However, the present results go further than the initial assumptions within ACT, by implicating effects of trait somatic anxiety on performance, not just worry (i.e., cognitive anxiety). Looking first to cognitive anxiety, there is more evidence and theoretical support for the effects of worry/cognitive anxiety on cognition (e.g., Edwards et al., 2015, 2017; Gustavson and Miyake, 2016; Mella et al., 2020) than for effects of somatic anxiety. Thus, it is proposed that the observed prediction of cognitive difficulties associated with trait cognitive anxiety may be due to the impact of anxiety on attentional systems. That is, worry occupies resources, bringing difficulty across cognitive processes (Eysenck et al., 2007; Berggren and Derakshan, 2013; Mella et al., 2020). Consequently, performance deficits across multiple cognitive domains are possibly being influenced by the changes in attention regulation (e.g., Bledowski et al., 2004; Arnell et al., 2007; Blair et al., 2007; Harada et al., 2013). Therefore, the present study provides support for ACT, suggesting that difficulties in attentional control arise from higher levels of trait anxiety (e.g., Edwards et al., 2015, 2017; Gawda and Szepietowska, 2016).

Trait somatic anxiety was found to significantly predict perceived cognitive difficulty specifically with language and verbal memory. While some recent evidence suggests that trait somatic anxiety can indeed negatively affect visual working memory (Spalding et al., 2021), spatial working memory (Vytal et al., 2013), and processing speed (Schoen and Holtzer, 2017), the present results are somewhat unexpected in the context of ACT (Eysenck et al., 2007). One possible explanation for this outcome is that trait somatic anxiety also demands attention. For example, trait somatic anxiety has been associated with greater internal focus at rest, potentially due to increased proprioception, that is, increased awareness of bodily sensations (Burdwood et al., 2016). Indeed, trait anxiety has been associated with greater monitoring of bodily signals (e.g., Ginzburg et al., 2014). There is also evidence that anxious somatic arousal is predicted by anxiety sensitivity (Vujanovic et al., 2007), which is the cognitive fear of anxiety and anxiety-related experiences. It is therefore possible that the self-report measure of trait somatic anxiety used in the present study also reflects underlying anxious cognitions. If individuals report experiencing somatic anxiety frequently at the trait level, this may reflect the attention they pay toward these experiences at the expense of attending to goal-relevant information.

Discrepancies between the results of previous studies regarding the effects of cognitive and somatic anxiety on cognition may have arisen due to the specific measure of anxiety employed. Differences may also have arisen from the specific cognitive outcomes assessed. Edwards et al. (2017) used the STICSA (Ree et al., 2008), the same measure used in the present study, and found effects of only cognitive anxiety on attentional control. However, they also examined a potential interaction effect between anxiety and situational stress (by threat of electric shock and ego-threat instructions). Spalding et al. (2021) also used the STICSA to assess the impact of anxiety on visual working memory, finding that while both dimensions of trait anxiety affected performance efficiency, only trait somatic anxiety predicted performance accuracy. Schoen and Holtzer (2017) used the Beck Anxiety Inventory (BAI; Beck et al., 1988), which, like the STICSA, is a measure of daily life, and found somatic, but not cognitive anxiety, to be associated with cognitive performance in older adults. Also examining performance in older adults, Mella et al. (2020) used the emotional and cognitive anxiety scale (EAEE; Beaudoin and Desrichard, 2009), which assesses state anxiety across cognitive and somatic dimensions, and found cognitive anxiety was the only predictor of performance between the two. Thus, those studies that have observed effects of cognitive anxiety have focused on anxiety at the state level, or its potential interaction with a situation-specific stress manipulation. By comparison, those that have found effects of somatic anxiety have focused on measuring anxiety at the trait level. The present results also focused on trait anxiety and found effects of both cognitive and somatic anxiety, albeit with the former having a more consistent relationship with cognition across age. Thus, when anxiety is measured as a relatively stable aspect of personality, both cognitive and somatic anxiety may negatively affect cognitive performance and subjective cognition.

### Limitations and Future Directions

Although the current study found age significantly moderated the effect of trait anxiety across cognitive domains, limitations and suggestions for future research should be considered. Firstly, the current study found significant relationships between each anxiety predictor and a number of the subjective cognitive difficulty outcomes. However, it cannot be assumed that these relationships are causal, in either direction. Indeed, it is possible that older adults who self-report greater cognitive difficulties, or objectively experience greater cognitive difficulties, in turn experience and self-report greater levels of anxiety. Future research should build upon this and previous studies exploring the potential interaction between anxiety and age on cognition by incorporating longitudinal and/or experimental methods. For example, previous studies of the effects of anxiety on cognition have compared performance under different levels of cognitive load (Vytal et al., 2012), or have accounted for individual differences in working memory capacity (Owens et al., 2012, 2014; Maloney et al., 2014). It would be beneficial to account for objective cognitive performance, and the extent to which this reflects subjective cognitive difficulties. This would help to clarify the extent to which subjective appraisals of cognitive difficulties reflect underlying objective difficulties, and in turn enable further understanding of how these difficulties may be related to anxiety.

Secondly, demographics mostly suggest that our sample reflects the general population, however, the majority of participants were female. There is evidence that women tend to report more symptoms of anxiety compared to men (Arcand et al., 2020). Although, this is also said to be due to females’ increased risk of anxiety disorders in general, compared to males (Balsamo et al., 2015). Therefore, future studies are encouraged to verify these results with a more representative sample regarding gender. Our sample also contained slightly more young adults than middle-aged and older adults. An equal distribution across age would therefore also be beneficial in future work.

Regarding our measures, these were limited in that processing speed, an important aspect of cognition affected by aging, could not be measured *via* the MASQ (Seidenberg et al., 1994). Also, as noted above, we used self-report questionnaires across all variables and the findings could be different when objective measures of cognitive abilities are included (e.g., Opdebeeck et al., 2019). Society tends to view older adults as declining in aspects of cognition such as memory, while some aspects such as knowledge and experience are preserved or improve (Hertzog et al., 2008). This may affect self-report responses and how especially older adults perceive their cognitive abilities relative to that of their peers or younger adults (e.g., Hertzog et al., 2008; Soubelet and Salthouse, 2011). Future research should therefore consider both subjective (e.g., self-report) and objective (e.g., lab-based or more real-world cognitive ability assessments) measures of cognitive functioning to overcome barriers associated with self-report, as well as to assess the relationship between self-reported cognitive abilities and observed cognitive performance, which is currently lacking in the literature (Herreen and Zajac, 2017). A useful application of this would be to determine why some previous research has shown effects of anxiety on objective measures of visual working memory, spatial working memory, and visual attention (e.g., Moser et al., 2012; Vytal et al., 2013; Spalding et al., 2021), yet effects of trait anxiety on subjective measures of visual-perceptual abilities were not observed presently. Furthermore, effects of anxiety on visual-spatial memory were inconsistent in the present research, in that a significant relationship was observed with trait cognitive but not trait somatic anxiety. Indeed, aspects of visual information processing and visual attention could potentially benefit from heightened anxiety in terms of enhanced visual detection of stimuli (e.g., Berggren et al., 2015; Minnick et al., 2020). Complex relationships across perceptual and cognitive domains of functioning should therefore benefit from further exploration with object measures and experimental manipulations. Although, it is important to recognize the value of the subjective measures used in the present study as providing an indication of how individuals experience cognitive difficulties in their everyday life. Objectively measuring performance under lab conditions tends to be limited in terms of ecological validity, or being relevant to a specific point in time. Thus, future research could usefully employ various methods across studies, assessing anxiety and cognition in everyday life and in the lab, both cross-sectionally and longitudinally.

### Conclusion

To conclude, the present study provided evidence for a moderating effect of age on the relationship between trait cognitive anxiety and perceived cognitive difficulty regarding the attention, verbal memory, visual-spatial memory, and language domains of cognition, but not for visual-perceptual ability. The current study also highlighted much less consistent moderating effects of age on the relationship between trait somatic anxiety and cognition, with the interaction observed only for verbal memory. Altogether, this research has demonstrated robust relationships amongst age, trait cognitive anxiety and subjective cognition, showing that across the adult lifespan, higher levels of specifically trait cognitive anxiety tend to be associated with greater subjective cognitive difficulties.

## Data Availability Statement

The raw data supporting the conclusions of this article will be made available by the authors, without undue reservation.

## Ethics Statement

The studies involving human participants were reviewed and approved by the School of Psychological Sciences and Health Ethics Committee, University of Strathclyde. The participants provided their written informed consent to participate in this study.

## Author Contributions

LN formulated the research, led development of the materials and data collection, and contributed to data analysis and drafting the manuscript. DS led the data analysis and drafting of the manuscript. KM and MM contributed to the development of materials, data collection, and drafting of the manuscript. All authors contributed to the article and approved the submitted version.

## Conflict of Interest

The authors declare that the research was conducted in the absence of any commercial or financial relationships that could be construed as a potential conflict of interest.

## Publisher’s Note

All claims expressed in this article are solely those of the authors and do not necessarily represent those of their affiliated organizations, or those of the publisher, the editors and the reviewers. Any product that may be evaluated in this article, or claim that may be made by its manufacturer, is not guaranteed or endorsed by the publisher.
